# Bioimpedance-Derived Membrane Capacitance: Clinically Relevant Sources of Variability, Precision, and Reliability

**DOI:** 10.3390/ijerph20010686

**Published:** 2022-12-30

**Authors:** Valene Garr Barry, Jasmine L. Chiang, Kaylan G. Bowman, Kristina D. Johnson, Barbara A. Gower

**Affiliations:** 1 Department of Obstetrics and Gynecology, Division of Clinical Research, School of Medicine in St. Louis, Washington University, St. Louis, MO 63108, USA; 2 Department of Obstetrics and Gynecology, School of Medicine, The University of Alabama at Birmingham, Birmingham, AL 35294, USA; 3 Department of Nutrition Sciences, School of Health Professions, The University of Alabama at Birmingham; Birmingham, AL 35294, USA

**Keywords:** membrane capacitance, bioimpedance, reliability, women, health status

## Abstract

Membrane capacitance (C_M_), a bioimpedance-derived measure of cell membrane health, has been suggested as an indicator of health status. However, there are few published data to support its use in clinical settings. Hence, this study evaluated clinically relevant sources of variation, precision, and reliability of C_M_ measurements. This longitudinal study included 60 premenopausal women. Sources of variability (e.g., demographics, body composition, serum measures, diet) were identified by stepwise regression. Precision and reliability were assessed by the coefficient of variation (CV), intraclass correlation coefficients (ICC), and technical error of the measurement (TEM) for intra-day (30 min apart) and inter-day measurements (7–14 days apart). Body composition, temperature, and metabolic activity were identified as sources of variability. C_M_ measurements had high precision (CV = 0.42%) and high reliability for intra-day (ICC = 0.996) and inter-day (ICC = 0.959) measurements, independent of menstrual cycle and obesity status. Our results showed that C_M_ measurements are sensitive to clinical factors and have high precision and reliability. The results of this study suggest that C_M_ is sufficiently reliable for health status monitoring in conditions with variation in body composition, metabolic activity, or body temperature among premenopausal women.

## 1. Introduction

There has been growing interest in using bioimpedance approaches to non-invasively transform the electrical properties of biological tissues into clinically useful information [1,2,3,4,5,6,7]. Bioimpedance is a technique that passes standard alternating current through the water- and electrolyte-rich tissues of the body and characterizes their electrical properties in terms of resistance (R) and reactance (Xc), which, respectively describe the opposition to the current and polarization of tissues at one or more measured frequencies [2]. The relationship between R and Xc can be used to inform on the health status of an individual through the bioimpedance measure membrane capacitance (C_M_), which is derived from modeling Xc versus R over a spectrum of frequencies. C_M_ reflects the health and integrity of the cell membranes within the path of the current [4,8,9]. Likewise, a similar and more heavily investigated measure is the phase angle, which is the ratio of Xc to R at a single frequency. Several studies have emerged showing that C_M_ and the phase angle could have prognostic value for a wide array of medical conditions, including end-stage diseases (e.g., renal disease, cardiac insufficiency, liver cirrhosis, sickle cell disease) and early-stage metabolic dysfunction (e.g., obesity, malnutrition, inflammation, metabolic syndrome, and insulin resistance) [10,11,12,13,14,15,16]. However, there are relatively few studies that have investigated C_M_ measurements for clinical or epidemiological use. 

Sources of variability or clinical determinants of C_M_ have not been thoroughly examined, and to the best of our knowledge, there are no published studies on the reliability of C_M_ measurements. Much of what is known about C_M_ comes from biomedical engineering studies, which are often difficult to interpret in a clinical context. Other information about C_M_ comes from extensive research on the phase angle, which has an imperfect relationship with C_M_ [8]. C_M_ may be more appropriate for health status monitoring in some conditions and more sensitive to early-stage metabolic dysfunction than the phase angle [2,3,8]. Additionally, C_M_ might be useful in conditions where the phase angle is unreliable, such as fluctuations in weight, hydration status, menstrual cycle hormones (premenopausal women), and obesity (e.g., a body mass index [BMI] > 34 kg/m^2^) [2,5,6,16,17]. However, further studies into C_M_ for clinical health status monitoring are needed.

Our group recently put forth that C_M_ could be used to identify premenopausal women with insulin resistance [13]. Therefore, in the present study, we aimed to determine whether C_M_ measurements are reliable enough to be used in clinical health status monitoring among premenopausal women. The primary objectives of this study were (1) to evaluate sources of variability in C_M_ considering clinically relevant factors, such as demographics, anthropometrics, body composition, and serum measures, and (2) to analyze the precision and reliability (intra-day and inter-day) of C_M_ measurements. The secondary objectives of this study were to evaluate the influence of the menstrual cycle and obesity status on C_M_ and the reliability of C_M_ measurements. 

## 2. Methods

### 2.1. Study Design and Participants

This was a prospective observational study of C_M_ in nondiabetic premenopausal women. Participants were recruited from the University of Alabama at Birmingham (UAB) campus and local communities around Birmingham, AL. Eligible participants were >18 years old with a BMI of 18.45–45 kg/m^2^. Exclusion criteria included weight fluctuations ± 4.54 kg in the prior six months; previous diagnoses of chronic or critical diseases, such as cancer or cardiovascular or cerebrovascular events; and use of potassium supplements, diuretics, or drugs that are known to regulate fluid balance. Those with contraindications for bioimpedance were excluded, including amputations, artificial joints, pins, plates, or other types of metal objects in the body; pacemaker or automatic defibrillator; or coronary stents or metal suture material in the heart. The UAB Institutional Review Board approved the study (protocol number 150701003), and all participants provided written and verbal consent before testing.

### 2.2. Study Schedule

Between October 2019 and March 2020, participants had a series of C_M_ measurements taken at two separate study visits. One visit occurred in the follicular phase (i.e., day 1–8) and another in the luteal phase (i.e., day 14–22) of the participant’s menstrual cycle. The order of visits was based on the participant’s availability, and women with irregular or absent cycles were assessed 7–14 days apart. Participants were asked to fast (no food or drink except plain water) for 10 h before testing, avoid drinking alcohol within 24 h of testing, avoid exercise or sauna use within 12 h of testing, and refrain from using hand or body lotion the morning of testing.

### 2.3. Anthropometrics

Anthropometric measurements were taken at each study visit, including height, weight, waist circumference, limb circumferences, and limb lengths. Height was measured with a wall-mounted stadiometer and was reported to the nearest 0.5 cm with an accuracy ± 2 mm. Weight was assessed using a standard digital scale that is accurate ± 0.01 kg and reported to the nearest 0.1 kg. Circumferences and lengths were measured with a flexible tape measure to the nearest 0.01 cm.

### 2.4. Bioimpedance Measurements

At each study visit, C_M_ was measured twice, 30 min apart. Participants were instructed to void their bladder before the first measurement of the day and were required to stand for approximately 10 min before each measurement. To compare C_M_ measurements between devices, both measurements were taken with participants standing in the anatomical position with a 30° angle between the arms and body and an approximate 45° angle between the legs [18]. Gel-backed electrodes were placed on the participant’s right hand/wrist and foot/ankle, with proximal and distal electrodes 5 cm apart. Electrodes were placed before the first measurement at each visit and were not removed/re-positioned for the second measurement. All measurements were performed by one of two trained study staff members, and the same staff person performed all intra-day measurements.

At each time point, bioimpedance measurements were performed on a bioimpedance spectroscopy device (SFB7; ImpediMed Ltd., Brisbane, QLD, Australia), which is a single-channel tetra-polar four-lead instrument that is connected via alligator clips to gel-backed electrodes on the participant’s right hand/wrist and foot/ankle. The spectroscopy device measured R and Xc of the right body side at 256 frequencies ranging from 3 kilohertz (kHz) to 1000 kHz. At each time point, three consecutive measurements were taken automatically. C_M_ measurements were obtained by fitting the collected R and Xc values from the right body side to the Cole-Cole Model using the BioImp^®^ spectroscopy software (Version 5.5.0.1, ImpediMed Ltd., Eight Mile Plains, Brisbane, QLD, Australia). 

### 2.5. Body Composition

Fat mass and lean mass for the total body, arms, legs, and trunk were measured by dual-X-ray absorptiometry (DXA) (iDXA, GE Healthcare Lunar, Madison, WI, USA). DXA assessments were performed during the follicular phase for women with normal menstrual cycles and during the first study visit for women with irregular or absent cycles. Urine pregnancy tests were administered before each participant underwent DXA scans.

### 2.6. Serum Measures

Following an overnight fast of at least 10 h, a blood sample was collected. Fasting sera were used to measure insulin, c-peptide, glucose, lipids, testosterone, progesterone, and estradiol by the UAB Nutrition Obesity Research Center and Diabetes Research Center Core Laboratory. Additional sera were used to measure serum electrolytes by the UAB Hospital Medical Laboratory.

Fasting glucose was determined using the glucose oxidase method on a SIRRUS analyzer (Stanbio Laboratory, Boerne, TX, USA). For glucose, the intra-assay coefficient of variation (CV) was 1.2%, and the inter-assay CV was 3.1%. Fasting insulin and c-peptide were assayed by immunofluorescence on an Automated Immunoassay Analyzer (AIA)-900 (TOSOH Bioscience, South San Francisco, CA, USA). For insulin and c-peptide, the respective intra-assay CVs were 1.5% and 1.7%, and the inter-assay CVs were 4.4% and 6.8%. The homeostatic model assessment of insulin resistance (HOMA-IR) was calculated by the equation [(fasting serum insulin (µU/mL) × fasting glucose (mg/dL))/22.5] [19].

Fasting total cholesterol, high-density lipoprotein cholesterol (HDL-C), and triglycerides were measured using a SIRRUS analyzer. For cholesterol, HDL-C, and triglycerides, the respective intra-assay CVs were 1.3%, 6.1%, and 1.1%, and inter-assay CVs were 4.3%, 6.6%, and 4.3%. Low-density lipoprotein cholesterol (LDL-C) was calculated using the Friedwald equation: total cholesterol—(triglyceride/5)—HDL-C [20]. Free fatty acids were assayed with “NEFA-C” reagents acquired from Wako Diagnostics (Richmond, VA, USA). The assay was modified to accommodate a reduced sample volume (10 μL) and the use of a microplate reader for the measurement of optical density at 550 nm. For FFA, the intra-assay CV was 7.4%, and the inter-assay CV was 3.7%.

Progesterone, testosterone, and estradiol were assessed by the fluorescent enzyme immunoassay (FEIA) method using the TOSOH AIA-900. For progesterone, testosterone, and estradiol, respectively, the inter-assay CVs were 2.26%, 10.21%, and 1.42%, and intra-assay CVs were 1.04%, 3.63%, and 2.31%.

The serum electrolytes, potassium (K^+^), sodium (Na^+^), and chloride (Cl^−^) were assessed using the Beckman Coulter Analyzer AU400 (Beckman Coulter Inc. Brea, CA, USA). For K^+^, Na^+^, and Cl^−^, the respective inter-assay CVs were 0.7%, 0.6%, and 0.6%, and intra-assay CVs were 0.6%, 0.3%, and 0.5%.

### 2.7. Temperatures

At the time of each bioimpedance measurement, an infrared thermometer (Model: JXB-178; Berrcom Medical Device Co., Ltd.; Guangzhou, China) was used to measure room temperature, peripheral body temperature (at the forehead), and skin temperature (at the back of the hand/wrist near electrodes). Temperatures were reported to the nearest 0.1°F and were accurate to ± 0.3 °F.

### 2.8. Other Variables

Medical history and medication use were assessed by questionnaires. Dietary intake was evaluated for the three days before each study visit using the smartphone-based calorie tracking app MyFitnessPal^®^ (Under Armor Inc., Baltimore, MD, USA), including total calories, carbohydrates, protein, fat, added sugar, and sodium. Each entry was reviewed with the study staff for completion and accuracy.

### 2.9. Statistical Analyses

Descriptive statistics were calculated with continuous variables expressed as mean ± standard deviation and categorical/dichotomous variables expressed as percentages (*n*). Pearson’s correlation coefficients were used to calculate the association of C_M_ with participant characteristics. Paired *t*-tests were used to compare participants’ clinical characteristics between the follicular and luteal phase study visits among women with normal menstrual cycles only.

Sources of variability in C_M_ were examined using stepwise linear regression models with standardized beta coefficients (STD-β). Possible predictors included in the model were demographics (e.g., age, race), body composition (e.g., weight, DXA fat mass), serum measures (e.g., glucose, lipids, electrolytes), medication use, dietary intake, and measurements conditions (e.g., temperatures, time of day). Sensitivity analyses were performed that included the bioimpedance-derived variables extracellular to intracellular water (ECW: ICW) ratio (i.e., hydration status) and total body water. Given that the inclusion of these factors significantly increased multicollinearity, these analyses were not included in the manuscript but are available in the Supplementary Materials.

The precision and reliability of C_M_ measurements from the spectroscopy device were measured in three ways. Multiple reliability statistics were calculated for comparison with existing bioimpedance reliability statistics and to provide clinically relevant reliability measures. First, the CV (standard deviation/mean x 100) was used to estimate the precision of three consecutive C_M_ measurements. A CV < 2% was considered acceptable for clinical use [21]. Second, intraclass correlation coefficient (ICC) estimates and their 95% confidence intervals were used to measure the reliability of two measurements taken 30 min apart (intra-day) and 7–21 days apart (inter-day, without accounting for the menstrual phase). ICC estimates less than 0.5 indicate poor reliability, estimates between 0.5 and 0.75 indicate moderate reliability, values between 0.75 and 0.9 indicate good reliability, and values greater than 0.90 indicate excellent reliability [22]. Third, the technical error of the measurement (TEM), relative TEM, and coefficient of reliability (Rcoeff) were also used to assess the reliability of intra-day and inter-day C_M_ measurements. TEM is equal to the standard deviation of differences between repeated measures, and lower TEM values indicate greater reliability. Relative TEM was calculated as TEM/mean measurement value × 100. Rcoeff is equal to 1-TEM^2^/(SD of measurements)^2^ and represents the proportion variance that is not attributable to measurement error (i.e., similar to the more clinical ICC). Higher Rcoeff values indicate greater reliability, with values above 0.95 considered acceptable, 0.8 considered sufficient, and values lower than 0.7 considered minimally acceptable measurement error [23].

The influence of the menstrual cycle on (a) C_M_ and (b) the reliability of C_M_ (i.e., differences in C_M_ measurements between visits/menstrual phases) was evaluated by paired *t*-tests among women with normal menstrual cycles only. The influence of obesity status on (a) C_M_ and (b) the reliability of C_M_ was evaluated by using analysis of variance (ANOVA) with least-squared means adjustment. Obesity was defined as having a BMI > 34 kg/m^2^ due to previous reports of increased error and lower reliability of bioimpedance beyond this BMI [3]. Sensitivity analyses (not shown) were performed using the conventional cutoff for obesity of 30 kg/m^2^, and the results were the same.

All appropriate assumptions were verified for each respective statistical test, including the assumptions of normality. All analyses were performed in SAS 9.4 (SAS Institute, Cary, NC, USA). Statistical significance was set at *p* < 0.05.

## 3. Results

### 3.1. Sample Characteristics

Of the sixty-three premenopausal women who enrolled in the study, 55 women completed both study visits, and eight women completed only one study visit. Three participants were excluded for non-compliance due to laboratory values that indicated either they had not fasted before the study visit or had type 2 diabetes. The 60 women who are included in the following analyses are described in Table 1. Repeated measurements are reported by menstrual cycle phase in Table 2 for the 52 women with normal menstrual cycles who completed both visits. Overall, participants had a mean age of 28 ± 8 years (range: 19–48 years) and a BMI of 28.9 kg/m^2^ (range: 18.81–47.33 kg/m^2^). Seventeen women had a BMI > 30 kg/m^2^, and eight women had a BMI > 34 kg/m^2^.

### 3.2. Sources of Variability for C_M_

The mean value of C_M_ was 1.60 ± 0.45 nF (range: 0.75–3.34 nF). A stepwise regression [*F* (9, 29) = 13.64, *p* < 0.0001; *R*^2^ = 0.81] showed that most of the variation in C_M_ was explained by DXA arm lean mass (STD-β = 0.76, *p* < 0.0001), height (STD-β = −0.36, *p* = 0.0006), HOMA-IR (STD-β = 0.43, *p* = 0.0014), skin temperature (STD-β = −0.53, *p* = 0.0003), body temperature (STD-β = 0.35, *p* = 0.003), and K^+^ (STD-β = 0.19, *p* = 0.0463) (Table 3). A separate regression model including bioimpedance-derived estimates of total body water and ECW: ICW ratio is shown in Supplementary Table S1.

### 3.3. The Precision and Reliability of C_M_

Triplicate C_M_ measurements (*n* = 235 sets) were taken approximately five seconds apart. The average CV for the three consecutive C_M_ measurements was 0.38% (range: 0.07%–0.72%), which is considered acceptable for clinical use.

Intra-day measurements (*n* = 116 pairs) were taken 32.94 ± 3.76 min apart (range: 15.50–39.50 min), and there was a mean difference of 0.05 ± 0.06 nF (range: −0.10, 0.22 nF). The ICC for intra-day measurements was 0.996, 95% CI: [0.993, 0.997], which indicates excellent reliability. The TEM, relative TEM, and Rcoeff for intra-day measurements were 0.05%, 3.43%, and 0.99, respectively, which is considered acceptable for clinical use.

Inter-day measurements (*n* = 55 pairs) were taken 14.9 ± 5.54 days apart (range: 5–33 days), and there was a mean difference of 0.03 ± 0.18 nF (range: −0.48, 0.41 nF). The ICC for inter-day measurements was 0.959, 95% CI: [0.929, 0.976]. The TEM, relative TEM, and Rcoeff for inter-day measurements were 0.13%, 7.93%, and 0.96, respectively.

### 3.4. The Influence of the Menstrual Cycle on C_M_

Paired *t*-tests showed that among women with normal menstrual cycles, C_M_ did not change significantly between the follicular and luteal phase [*t* (52) = 1.79, *p* = 0.079]. There was a mean menstrual phase difference in C_M_ of 0.04 ± 0.18 nF (range: −0.41, 0.48).

### 3.5. The Influence of Obesity Status on the Reliability of C_M_

In general, C_M_ was significantly higher in women with a BMI > 34 kg/m^2^ than in women with a BMI < 34 kg/m^2^, 2.10 ± 0.55 nF versus 1.52 ± 0.31 nF, respectively (*p* < 0.0001). Women with a BMI > 34 kg/m^2^ did not have significantly different inter-day (*p* = 0.78) or menstrual cycle phase (*p* = 0.53) differences in C_M_.

## 4. Discussion

The bioimpedance marker C_M_ has recently been put forth as a potential clinically useful indicator of health and disease for multiple conditions [1,2,3,4,5,6,7]. However, the proposition that bioimpedance approaches can be clinically useful for health status monitoring remains contentious due to (a) a generally poor understanding of bioimpedance and its underlying mechanisms; (b) an inability to differentiate between bioimpedance for body compositions versus health status monitoring; and (c) a lack of published clinical method validation studies [24]. Hence, the purpose of this study was to provide data regarding clinically relevant sources of variability, precision, and reliability of C_M_ measurements among premenopausal women.

### 4.1. Sources of Variability

In this study, C_M_ values ranged from 0.75 nF to 3.34 nF with an average of 1.60 nF, which is consistent with previously reported C_M_ values for young, healthy women [13,25]. As expected, the majority (i.e., 61%) of the variability in C_M_ was explained by the body composition variables DXA arm lean mass and height. Supplemental models, which were not included due to concerns for multicollinearity, showed that the ECW: ICW ratio, which is essentially hydration status, and DXA arm lean mass alone accounted for 50% of the variability C_M_. This finding is consistent with current views that C_M_ is a function of the volume of the body cell mass, body water, and the distribution of water between intracellular and extracellular spaces [8,9]. This finding also aligns well with proposals to use C_M_ for clinical monitoring of conditions that are characterized by variation in the body cell mass or body water, including liver cirrhosis, renal failure, and cardiac insufficiency [8].

The third strongest explanatory factor for variability in C_M_ was HOMA-IR, a surrogate measure of insulin resistance that has been previously associated with C_M_ [13,26]. HOMA-IR, serum K^+^, cholesterol, and progesterone accounted for 10% of the variability of C_M_. Collectively these factors may reflect the influence of the cell membrane’s metabolic activity (e.g., substrate transport, insulin-mediated processes) on C_M_ because each of these factors is intimately involved in insulin-mediated metabolism or glucose homeostasis in the skeletal muscle, which is the primary site that is characterized in bioimpedance [27,28,29,30]. These findings are consistent with recent evidence that C_M_ may be influenced by disturbances to metabolic activity independent of changes in hydration or body cell mass in conditions such as inflammation, high-fat diets, metabolic syndrome, and insulin resistance [11,12,13,14,31,32].

Another 10% of the variability in C_M_ was explained by temperature measurements for the skin, peripheral body (e.g., forehead), and room. Temperature is well known to influence bioimpedance measurements [3,33,34,35]. Prior studies have demonstrated that bioelectrical resistance is greater in cold temperatures [33], which suggests that derived measures such as C_M_ might be lower in colder temperatures. However, most studies that investigate C_M_ or related bioimpedance terms in a clinical context did not report any temperature measurements. The prevailing notion is that bioimpedance measurements are largely influenced by the temperature of the skin, which is inextricably linked to both body temperature and ambient temperature through changes in blood flow and plasma volume that occur as the body adapts to changes in the internal or external environment [33,36]. Body temperature, and subsequently skin temperature, is also influenced by several disease states, including both infectious (e.g., viral infections) and metabolic disease (e.g., obesity), as well as several medications [37,38]. Furthermore, there are known differences in the regulation of body temperature between men and women, with women exhibiting variations in body temperature resultant of sex hormones and the menstrual cycle [39]. Therefore, these findings indicate that clinical applications of C_M_ should consider the appropriate temperatures in their interpretation of results.

Collectively the clinically relevant variables that were examined in this study accounted for 81% of the variability in C_M_. However, there are several technical factors known to influence bioimpedance measurements that were not addressed in this manuscript. Of note are the well-documented sources of technical variability (error), including lead placement, postural changes, electrode selection, and modeling constraints, to name a few [6,8,40,41,42,43]. Hence, Studies of derived bioimpedance parameters such as C_M_ should observe all potential sources of variability upon clinical use.

### 4.2. Precision/Reliability

C_M_ measurements had high precision and high reliability for both intra-day and inter-day measurements according to all reported reliability statistics; ICC and Rcoeff > 0.9, TEM < 0.2%. It is noteworthy that the relative TEM for inter-day C_M_ measurements was 7.93%, which may be higher than ideal for clinical use in conditions where there is a smaller difference between groups. However, rather than error, this may actually reflect variability in C_M_ caused by menstrual cycle fluctuations, which are discussed further below. This finding aligns well with published reliability statistics for other bioimpedance measures. Among young women, intra-day (i.e., 15 min apart) phase angle measurements had a TEM ranging from 0.10% to 0.60%, depending on measurement frequency. Comparable inter-day reliability statistics for the phase angle were unavailable. However, the inter-day TEM for R and Xc were 23.8% and 11.0%, respectively, in young women [30,44]. It is noteworthy that care was taken to mitigate the previously mentioned sources of technical error, such as lead placement, postural changes, electrode selection, and modeling inconsistencies. Such errors may still be present, however small, and clinical applications of C_M_ should observe these potential sources of error. Nevertheless, the results of this study suggest that C_M_ may be more reliable than other bioimpedance measures in premenopausal women. This study adds precision and reliability data for C_M_ to the literature and supports the suitability of using C_M_ for clinical monitoring in premenopausal women.

### 4.3. Menstrual Cycle

The menstrual cycle exerted a slight but non-significant effect on C_M_ measurements. We found a 2.5 ± 11.5% increase in C_M_ from the follicular to the luteal phase. Previous authors have noted that hormonal and physiological changes associated with the menstrual cycle may influence raw bioimpedance terms, including R and Xc, and the accuracy of body composition predictions [30,45,46]. However, we found no published data comparing C_M_ or the phase angle across the menstrual cycle. This finding suggests that C_M_ measurements used for health status monitoring may not need to be standardized measurements to occur in a specific menstrual cycle phase. However, given that there was a normal distribution of differences, with some women experiencing larger differences than others, the menstrual cycle phase should still be considered, especially in longitudinal analyses.

### 4.4. Obesity Status—BMI > 34 kg/m^2^

The reliability of C_M_ measurements did not differ according to obesity status. Although C_M_ was significantly higher among women with a BMI > 34 kg/m^2^, we found that the intra-day, inter-day, and menstrual phase differences in C_M_ measurements were similar between those with and without obesity, using both the bioimpedance-specific cutoff of 34 kg/m^2^ and the conventional cutoff of 30 kg/m^2^ (not shown). This finding was somewhat contrary to our expectations because it has been noted that bioimpedance-based approaches are unreliable for body composition and fluid estimation among women with obesity, and it has been questioned whether bioimpedance-based health status monitoring is similarly erroneous in this population [47,48,49,50,51]. Further, women with obesity, especially severe obesity, tend to have greater fluctuations across the menstrual cycle in factors that could influence bioimpedance and C_M_ measurements, including body weight, hydration, electrolyte balance, hormones, and dietary intake [37,38,39,47,52,53]. Nevertheless, our results suggest that C_M_ measurements should be equally reliable in women with obesity. These findings also indicate that C_M_ measurements used for health status monitoring should account for either body composition or BMI with either stratification or statistical adjustments.

### 4.5. Limitations

The results of this study should be interpreted considering the following limitations. Regarding variability, this study did not collect direct measurements of total body water or hydration status. We performed supplemental analyses using bioimpedance-derived measures of body water and ECW: ICW ratio. However, these models included a high degree of multicollinearity and thus violated the assumptions of the statistical models. Regarding precision and reliability, we were unable to completely differentiate between inter-day and menstrual cycle phase differences in C_M_ because there were only two assessment days. Additionally, we did not account for differences in menstrual cycle length between participants in our analyses.

### 4.6. Future Directions

Further investigation of C_M_ is needed. Regarding variability, future studies should continue to examine C_M_ in the context of different populations (e.g., male sex, children, and elderly populations) and specific clinical conditions. Additionally, studies that investigate C_M_ should report sources of variability to determine the reproducibility of results. Likewise, studies should observe the effects of known technical sources of error. Regarding precision and reliability, future studies might compare multiple inter-day measurements within the same menstrual cycle and identify an optimal time for measurements. Additionally, precision and reliability statistics should be included in studies investigating C_M_ so that reliability statistics for different devices and clinical conditions can be represented in the literature.

## 5. Conclusions

This study performed a critical examination of C_M_ among relatively healthy premenopausal women. The major findings of this study are that (1) variability in C_M_ is associated with body composition, metabolic activity, and temperature; (2) C_M_ measurements have a high level of precision; and (3) C_M_ measurements have high inter- and intra-day reliability. The novelty and importance of this study is that we placed C_M_ into a clinical context by providing clinically relevant sources of variability that may be important for clinicians and epidemiologists who might wish to use C_M_ in their research. Further, the high level of precision and reliability that was observed in this study, especially in women with obesity, indicates that C_M_ might be a robust indicator for clinical health status monitoring in premenopausal women. Our results support existing studies claiming that bioimpedance and C_M_ may be useful for clinical monitoring of conditions where there are significant disturbances to body composition, hydration, or metabolic activity. Therefore, this study also adds to the literature clinically relevant information about C_M_ that can be used to critically evaluate C_M_ studies and that may support further research into C_M_.

## Figures and Tables

**Table 1:** Descriptive Characteristics and Pearson’s Correlation Coefficients for the Relationship with Membrane Capacitance (*n* = 60).

	Mean ± SD	Range [Min–Max]	Correlation with C_M_ (|r|)
***Demographics***			
Age, years	28.80 ± 7.87	19.0–48.0	0.10
Race/Ethnicity, % (*n*)			
Asian	4.9 (3)		
Black	41.0 (25)		
Hispanic	11.5 (7)		
White	42.6 (26)		
***Body Composition***			
BMI, kg/m^2^	28.84 ± 7.67	18.81–47.33	0.57 ***
BMI Class, % (*n*)			
18.5–24.9	39.3 (24)		
25.0–29.9	32.8 (20)		
30.0 +	27.9 (17)		
Lean mass, DXA, kg	44.60 ± 7.32	31.26–71.34	0.65 ***
Fat mass, DXA, kg	31.22 ± 15.45	12.03–75.07	0.45 **
Percent Fat, DXA, %	39.22 ± 8.26	22.42–55.75	0.32 *
***Menstrual Cycle Hormones***			
Menstrual Cyclicity, % (*n*)			
Normal Cycle	86.7 (52)		
Irregular/Absent Cycle	13.3 (8)		
Testosterone, ng/dl	45.09 ± 23.98	10.00–112.90	0.23 *
Estradiol, pg/dl	95.71 ± 78.79	25.00–359.00	0.14
Progesterone, ng/ml	3.89 ± 6.96	0.12–29.01	−0.28 *
***Serum Electrolytes***			
Potassium, mmol/L	4.36 ± 0.34	3.80–5.40	0.38 *
Sodium, mmol/L	138.59 ± 2.04	134.00–143.00	−0.05
Chloride, mmol/L	103.19 ± 2.00	99.00–107.00	−0.03
***Bioimpedance***			
Membrane Capacitance, nF	1.58 ± 0.47	0.75–3.34	

Significance of correlations indicated by asterisks:*** *p* < 0.0001; ** *p* < 0.01; * *p* < 0.05. Abbreviations: DXA—dual-X-ray absorptiometry; nF—nanoFarads. Grey-filled box—intentionally left blank.

**Table 2:** Comparison of Clinical Characteristics by Menstrual Cycle Phase.

	Follicular Phase (*n* = 53)	Luteal Phase (*n* = 51)	*p*
	Mean ± SD	Mean ± SD	
Menstrual Cycle Day	5 ± 4	18 ± 4	**<0.0001**
***Body Composition***			
Weight, kg	76.42 ± 20.18	75.39 ± 19.09	0.22
Waist circumference, cm	85.79 ± 15.24	83.45 ± 16.50	0.59
BMI, kg/m^2^	28.40 ± 7.04	28.16 ± 6.59	0.76
Systolic BP, mmHg	118.74 ± 10.85	117.06 ± 10.56	0.28
Diastolic BP, mmHg	79.21 ± 7.58	77.31 ± 7.00	**0.03**
***Serum Measures***			
Insulin, fasting, uU/mL	10.80 ± 6.11	11.91 ± 9.40	0.19
Glucose, fasting, mg/dL	97.71 ± 32.63	97.32 ± 25.80	0.75
HOMA-IR	2.63 ± 1.65	2.92 ± 2.60	0.24
Total cholesterol, mg/dL	195.39 ± 40.98	187.53 ± 33.77	**<0.01**
Triglycerides, mg/dL	76.80 ± 36.12	78.28 ± 37.51	0.59
HDL-C, mg/dL	71.76 ± 14.09	70.43 ± 12.95	0.36
LDL-C, mg/dL	108.27 ± 33.09	101.42 ± 28.94	**<0.01**
Free fatty acids, mg/dL	0.66 ± 0.31	0.57 ± 0.24	**0.05**
***Menstrual Cycle Hormones***			
Testosterone, ng/dL	38.69 ± 16.99	42.95 ± 22.77	**0.04**
Estradiol, pg/dL	75.79 ± 60.25	112.33 ± 78.61	**0.03**
Progesterone, ng/mL	2.35 ± 5.40	4.18 ± 6.06	0.33
***Serum Electrolytes***			
Potassium, mmol/L	4.37 ± 0.27	4.32 ± 0.34	**0.05**
Sodium mmol/L	138.37 ± 2.13	138.40 ± 2.79	0.69
***Bioimpedance Measures***			
Total body water	37.02 ± 6.91	36.81 ± 6.85	0.33
ECW: ICW ratio	0.71 ± 0.04	0.71 ± 0.03	0.32
Membrane capacitance, nF	1.64 ± 0.43	1.61 ± 0.39	0.48
***Temperatures***			
Body temperature, °F	97.78 ± 0.25	97.76 ± 0.28	0.39
Room temperature, °F	71.38 ± 1.50	71.41 ± 1.74	0.74
Skin temperature, °F	85.60 ± 3.46	85.18 ± 3.96	0.74
***Dietary Intake***			
Energy intake, kcal	1564.38 ± 434.93	1508.34 ± 427.41	0.21
Sodium, mg	2126.29 ± 995.62	2102.36 ± 976.29	0.89
Added sugar, g	57.81 ± 34.61	57.57 ± 33.97	0.53

*p*-value for paired *t*-test comparison between menstrual phases. Bold values indicate significant *p* < 0.05.

**Table 3:** Stepwise Selection of Sources of Variability in Membrane Capacitance (C_M_).

Model: *F* (9, 29) = 13.64, *p* < 0.0001; *R*^2^ = 0.81; Adjusted *R*^2^ = 0.75							
Outcome *Variable:* C_M_							Final Model Effects
Step	Variable Added	Partial *R*^2^	Model *R*^2^	*F* Change	*p*	STD-β	*p*
**1**	DXA arm lean mass, kg	0.50	0.50	37.65	<0.0001	0.76	<0.0001
**2**	Height, cm	0.11	0.61	10.20	0.0029	−0.36	0.0006
**3**	HOMA-IR	0.05	0.66	4.94	0.0329	0.43	0.0014
**4**	Skin temperature, °F	0.04	0.70	4.21	0.0481	−0.53	0.0003
**5**	Body temperature, °F	0.03	0.73	3.21	0.0822	0.35	0.003
**6**	Room temperature, °F	0.03	0.76	3.86	0.0581	0.27	0.0389
**7**	Potassium, serum, mmol/l	0.02	0.78	3.05	0.0904	0.19	0.0463
**8**	Total cholesterol, mg/dl	0.02	0.79	2.39	0.1328	0.14	0.1333
**9**	Progesterone, ng/ml	0.02	0.81	2.35	0.1362	−0.13	0.1362

Abbreviations: STD-β—standardized beta coefficient; DXA—dual-X-ray absorptiometry.

## Data Availability

Please contact the corresponding author (valene@wustl.edu) for inquiries regarding data availability.

## Supplementary Materials

The following supporting information can be downloaded at: https://www.mdpi.com/article/10.3390/ijerph20010686/s1, Table S1: Stepwise Selection of Sources of Variability in Membrane Capacitance (CM)—Including Bioimpedance Estimates of Body Water and Hydration.
