# Supplementary material for: Bioimpedance-Derived Membrane Capacitance: Clinically Relevant Sources of Variability, Precision, and Reliability
Source: Int J Environ Res Public Health. 2022 Dec 30;20(1):686. doi: 10.3390/ijerph20010686 (PMC9819400; doi:10.3390/ijerph20010686)
Supplement: Supplementary file 1 [file ijerph-20-00686-s001.zip › ijerph-2060220-supplementary.pdf]

Supplemental Table S1. Stepwise Selection of Sources of Variability in Membrane Capacitance (C_M) – Including Bioimpedance Estimates of Body Water and Hydration

Model: $F(12,38) = 63.54$, $p < 0.0001$; $R^2 = 0.97$; Adjusted $R^2 = 0.95$

Outcome Variable: C_M							Final Model Effects	
**Step**	**Variable Added**	**Variable Removed**	**Partial R^2**	**Model R^2**	***F* Change**	**P**	**STD-β**	**P**
1	ECW:ICW ratio		0.59	0.59	52.58	<.0001	-0.70	<.0001
2	DXA arm lean mass, kg		0.24	0.82	47.75	<.0001		
3	Dietary energy intake, kcal		0.03	0.86	8.44	0.0063	-0.09	0.0333
4	HOMA-IR		0.03	0.88	7.80	0.0085	2.47	0.0001
5	Insulin, fasting, uU/ml		0.01	0.89	3.34	0.0769	-2.09	0.0005
6	Waist circumference, cm		0.01	0.91	3.69	0.0638	0.45	0.0081
7	Glucose, fasting, mg/dl		0.03	0.93	12.30	0.0014	-0.55	0.0087
8	Skin temperature, °F		0.01	0.94	4.39	0.0446	-0.14	0.0191
9		DXA Arm Lean Mass, kg	0.00	0.94	1.83	0.1860		
10	Potassium, serum, mmol/l		0.01	0.95	5.13	0.0309		
11	Thigh circumference, cm		0.01	0.95	2.99	0.0942	0.06	0.1057
12	Visceral fat mass, g		0.01	0.96	3.50	0.0719	-0.32	0.0099
13	Leg length, cm		0.00	0.96	3.40	0.0763	0.14	0.0024
14		Potassium, serum, mmol/l	0.00	0.96	0.65	0.4262		
15	Race/ethnicity		0.00	0.96	2.57	0.1208	0.08	0.0813
16	Progesterone, ng/ml		0.00	0.97	2.29	0.1420	0.07	0.0877
17	Dietary fat intake, g		0.00	0.97	2.46	0.1293	0.26	0.1293

Grey-filled boxes –variable was not included in the final model.

Abbreviations: STD-β – standardized beta coefficient; ECW: ICW ratio – extracellular to intracellular water ratio (i.e., hydration status); DXA– dual-x-ray absorptiometry.
